# The Effects of Altitude on Concussion Incidence in the 2013-2017 National Hockey League Seasons

**DOI:** 10.7759/cureus.2681

**Published:** 2018-05-24

**Authors:** Ryan Adams, Halley P Kaye-Kauderer, Syed Haider, Akbar Y Maniya, Stanislaw Sobotka, Tanvir F Choudhri

**Affiliations:** 1 Neurosurgery, The Icahn School of Medicine at Mount Sinai, New York, USA; 2 Surgery, Montefiore Medical Center, New York, USA

**Keywords:** concussion, head trauma, head trauma, environmental effects, high altitude, national hockey league, concussion incidence, concussion injury prevention

## Abstract

Background and objective

The high incidence of traumatic brain injuries during contact sports has necessitated the need for further research pertaining to their implications and possible mitigation. Despite increasing attention to sports-related concussions, there is still a striking lack of detail pertaining to the environmental factors that contribute to their occurrence. One environmental condition that has yet to be considered is altitude. Altitude cannot be readily adjusted, yet can still impact quality of play and concussion incidence. The current body of published evidence evaluating environmental effects on concussion is divided on the degree to which altitude mitigates concussion incidence. We aim to systematically compare the prevalence of concussions that occur at high and low altitude utilizing 1000 feet (304.8 meters) as a cut-off marker for high altitude. Our research also takes a novel approach utilizing average games missed as a proxy for concussion severity. We hope to use this analysis to shed light on the implication of altitude on concussion incidence.

Methods

Individual player data on concussion incidence were retrospectively acquired for the 2013-2017 National Hockey League (NHL) seasons utilizing FOX Sports Injury tracker. NHL season schedules were acquired through the online source “Hockey Reference.” In order to establish cutoff criteria for high vs low altitude we adopted 1000 feet (304.8 meters) as high-low altitude cutoff. We also evaluated our data utilizing a previously published high-low altitude cutoff of 644 feet (196.3 meters). Specific altitudes of each NHL arena were derived from “elevationmap.net”. One caveat to our data collection was the striking lack of publicly available data pertaining to the concussions sustained by each NHL team. Data was analyzed utilizing SAS programing.

Results

Out of the 5281 games included in our data set, we documented a total of 133 concussions which occurred in 125 games through the 2013-2017 NHL seasons. We noted an increase in concussion reporting in the most recent 2016-2017 NHL season compared to the previous 2013-2016 seasons. Effect of altitude variance on concussion rate was evaluated utilizing 644 and 1000 ft as the low-high altitude split. We defined each variance by where the team is based at compared to where the game was played. This produced four distinct categories: 1) low-low altitude, 2) low-high altitude, 3) high-low altitude, and 4) high-high altitude. We noted a significant difference in concussion rate when teams based at high altitude above 1000 ft travel to play at low altitude; this trend was non-significant at 644 ft. The results of the average games missed analysis demonstrated that teams that play above 1000 feet had fewer games missed per concussion compared to teams that are based at a low altitude.

Conclusions

Though underreported in the total number of concussions in the 2013-2017 NHL seasons, our data suggests that teams who are based at a high altitude (>1000ft) experience a reduction in mean concussion rate when traveling to play at a lower altitude. Our data also indicated a reduction in average games missed post-concussion for teams based at a higher altitude. It is our goal that our findings here contribute to the larger discussion about concussion incidence and can be applied to other sports leagues and activities to mitigate their dangerous effects.

## Introduction

In the past few years, there has been an increase in attention to both the prevalence and the short and long-term consequences of concussions. While the National Football League (NFL) has received lots of attention, focus is now shifting to other leagues like the National Hockey League where there is constant player-to-player contact as well as collisions with the perimeter boards. Due to increased awareness and research of traumatic brain injury, there have been several advances in concussion injury prevention. For instance, more research has begun on head-impact biomechanics, rules have been changed to limit injury exposure, equipment viability has been explored, and nutritional supplementation has been added to many player's diets [1, 2].

The popularization of traumatic brain injuries and their connection to contact sports have also led to a rise in research pertaining to their implications and possible mitigation. A report published in the Canadian Medical Association Journal examined physician reports from the 1997-2004 NHL seasons. They noted a total of 559 concussions during regular-season games. This implied a concussion rate of 5.8 for every 100 players, or an estimated 1.8 concussions per 1,000 player-hours [3]. A separate study by Wennberg et al. demonstrated that there has been a gradual increase in the average number of games missed per concussion during the 1997-2008 seasons. This study evaluated time lost from play data that was available for 310 concussions during the first five seasons of the study period and for 288 concussions during the last five seasons. The mean number of missed games per concussion during the last five seasons was significantly greater than during the first five seasons 12.9 ± 17.8 vs. 8.3 ± 13.1 [4].

Despite increasing attention to sports-related concussions, there is still a striking lack of detail pertaining to the environmental factors that contribute to their occurrence. The NHL presents itself as a uniquely interesting setting to study these factors since it mandates specifics on ice temperatures, humidity, and dew points. By doing so, the league aims to reduce environmental variance across different arenas to maximize the ice surface for game quality and safety [5]. However, one environmental condition that has yet to be considered is altitude. Unlike the other environmental factors, altitude cannot be readily adjusted, yet can still impact quality of play and concussion incidence. It has been postulated that a higher altitude increases cerebral blood flow, which in turn causes venous blood engorgement and an increased intracranial pressure. This leads to slight brain swelling and a seemingly tighter fit between the brain and skull to decrease brain sloshing and reduce concussive events [6]. Turner et al. (2012) demonstrated that attenuating the slosh effect in rats by compressing jugular venous outflow during acceleration/deceleration head injury reduced histologic markers of brain injury by up to 65%. However, the exact effect of altitude on concussion incidence still remains unknown, and research has yielded conflicting data on its impact [7].

While studies have begun to examine how environmental conditions like altitude affect concussion incidence in high-profile sports leagues, many questions still remain. The current body of published evidence evaluating environmental effects on concussion is divided on the degree to which altitude mitigates concussion incidence. Supporting evidence for the benefits and detriments of altitude has been proposed by both sides of the spectrum from the high school level up to professional tiers of competition [1, 5, 8-10]. One study even noted that higher altitude is unlikely to reduce risks for sports concussions utilizing a physiological perspective [11]. In response to this divided body of literature our study aims to provide clarification on this issue and to further elucidate the effects of altitude on mean concussion rates during four NHL regular seasons from 2013 to 2017. We aim to systematically compare the prevalence of concussions that occur for the home based and visiting teams utilizing 1000 feet (304.8 meters) as a marker for high altitude. Our research also takes a novel approach utilizing average games missed as a proxy for concussion severity comparable at low and high altitude. We hope to use this analysis to shed light on the implications of altitude on concussion incidence and to better understand potential protective or detrimental effects conferred from conditioning at high or low altitudes.

## Materials and methods

Data collection

One caveat to our data collection was the striking lack of publicly available data pertaining to the concussions sustained by each NHL team. We noted an under-reporting of concussions in our data set on a season to season basis compared to similar papers evaluating concussions in the NHL.

Data on concussion incidence for the 2013-2017 National Hockey League seasons was collected utilizing FOX Sports Injury tracker. After cross-referencing and comparing publicly available NHL injury reports, our research group deemed FOX Sports Injury tracker to be the most reliable and consistent across seasons. Only injuries specifically diagnosed as concussions during the regular and postseason were utilized in our data set. A Google search on the reported injury was performed in order to correlate the concussion to the correct game in which the player sustained it. This step was imperative in establishing the altitude at which the concussion occurred. NHL season schedules were acquired through the online source “Hockey Reference.” There were a total of 1230 regular season games per season we analyzed for a total of 4,920 games. Total postseason games varied season to season based upon how quickly teams advanced through the seven game series to the Stanley Cup Final. Concussions sustained during team practices and preseason contests were not accounted for in our data set to control for inconsistent reporting.

In order to establish cutoff criteria for high vs low altitude we adopted 1000 feet (304.8 meters) as high-low altitude cutoff. Data analysis was also conducted utilizing a previously published high-low cutoff of 644 feet (196.3 meters) for comparison [8]. Specific altitudes of each NHL arena were derived from “elevationmap.net” utilizing the specific street address of the arena to acquire the most exact altitude. 

Our dataset does not account for the current 2017-2018 NHL expansion with the addition of a Las Vegas team to the league.

Data analysis

Data was analyzed utilizing SAS programing (SAS Institute, Cary, NC). In addition to descriptive statistics, Fisher's exact tests, Welch’s two tailed t-tests, and correlation tests were used. An alpha level < 0.05 was considered significant for all tests. The mean concussion rate for teams based at a low altitude and traveling to play at a high altitude was compared to teams based at a high altitude traveling to playing at a low altitude. Low to low as well as high to high altitude games were also analyzed for concussion incidence.

## Results

**Figure 1:**
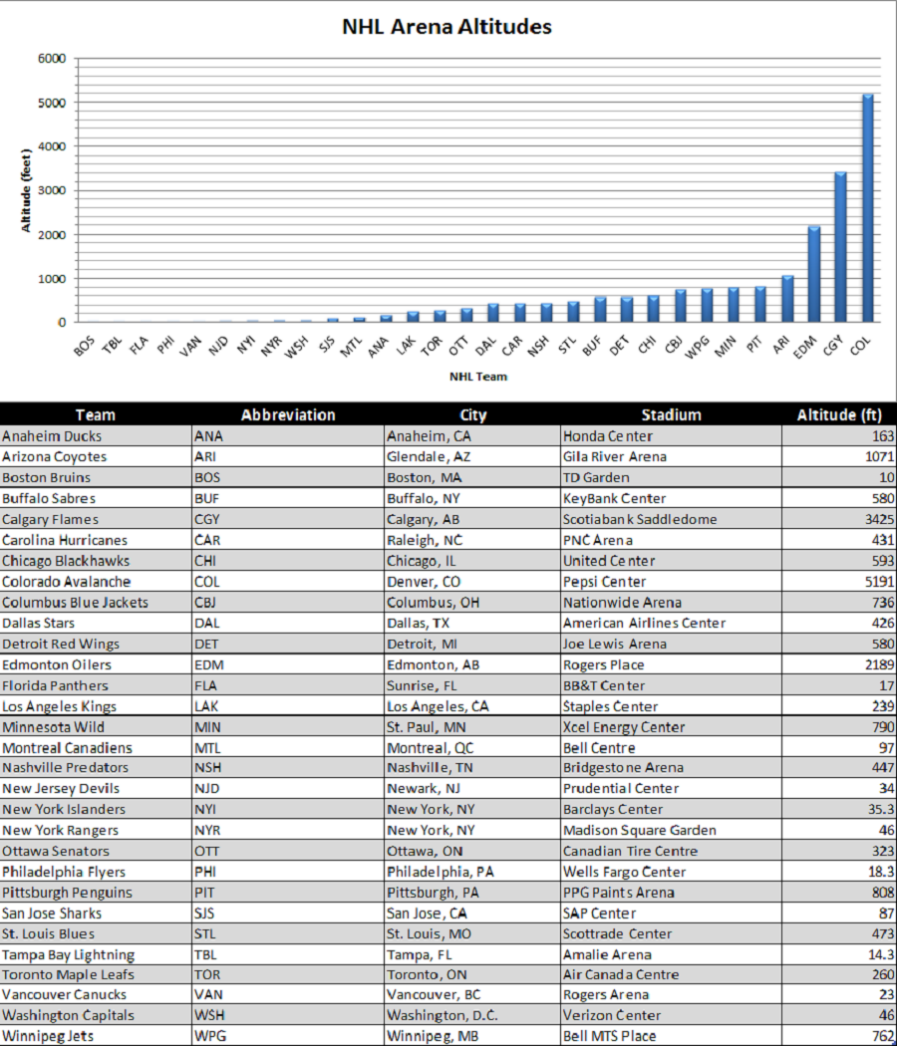
NHL Arena Altitudes NHL teams participating in the 2013-2017 seasons. Altitude (ft.) and location of each NHL arena approximated using the street address. Altitude values acquired utilizing elevationmap.net. Arena presented is representative of where the team played for the 2013-2017 seasons.

The altitudes of all NHL team arenas in the 2013-2017 seasons were documented, and four of the 30 total teams were based above the 1000 feet (304.8 meters) high-low altitude cutoff (Figure 1). It was noted that 11 of 30 teams were based less than 100 feet (30.48 meters) in altitude. The remaining 15 teams spanned in altitude from 101-999 feet above sea level throughout the United States and Canada.

Out of the 5281 games included in our data set, we documented a total of 133 concussions that occurred in 125 games through the 2013-2017 NHL seasons (Table 1). Aside from the variance in altitude, all other environmental factors such as indoor temperature, humidity, and dew point are mandated to be within a certain range for game time by the NHL. We noted an increase in concussion reporting in the most recent 2016-2017 NHL season compared to the previous 2013-2016 seasons (Table 1). This may be a product of increased attention to concussion identification and protocol.

**Table 1:** Demographic Data of Included Team Games Demographic analysis of concussion incidence on a season to season basis and home vs. away perspective. Indoor environmental factors are mandated to be within specific ranges during game time by the NHL (3), this implicates altitude as the most variable environmental factor on concussion incidence within our data set.

Demographics Data of Included Team Games (N = 5281)		
Variable	n	%
Total 2013-2014 Season Concussions	36	27
Total 2014-2015 Season Concussions	27	20
Total 2015-2016 Season Concussions	24	18
Total 2016-2017 Season Concussions	46	35
Total Number of Concussions	133	100
Home Team Concussions	71	53
Away Team Concussions	62	47
Total Games with Concussion	125	2
Total Games without Concussion	5156	98
Mean-Game Day Temperature Range consistent (°F)	60-65	100
On Ice Temperature Range (°F)	18-24	100
Mean-Game Day Humidity	35-45	100
Mean-Game Day Dew Point	32	100

**Figure 2:**
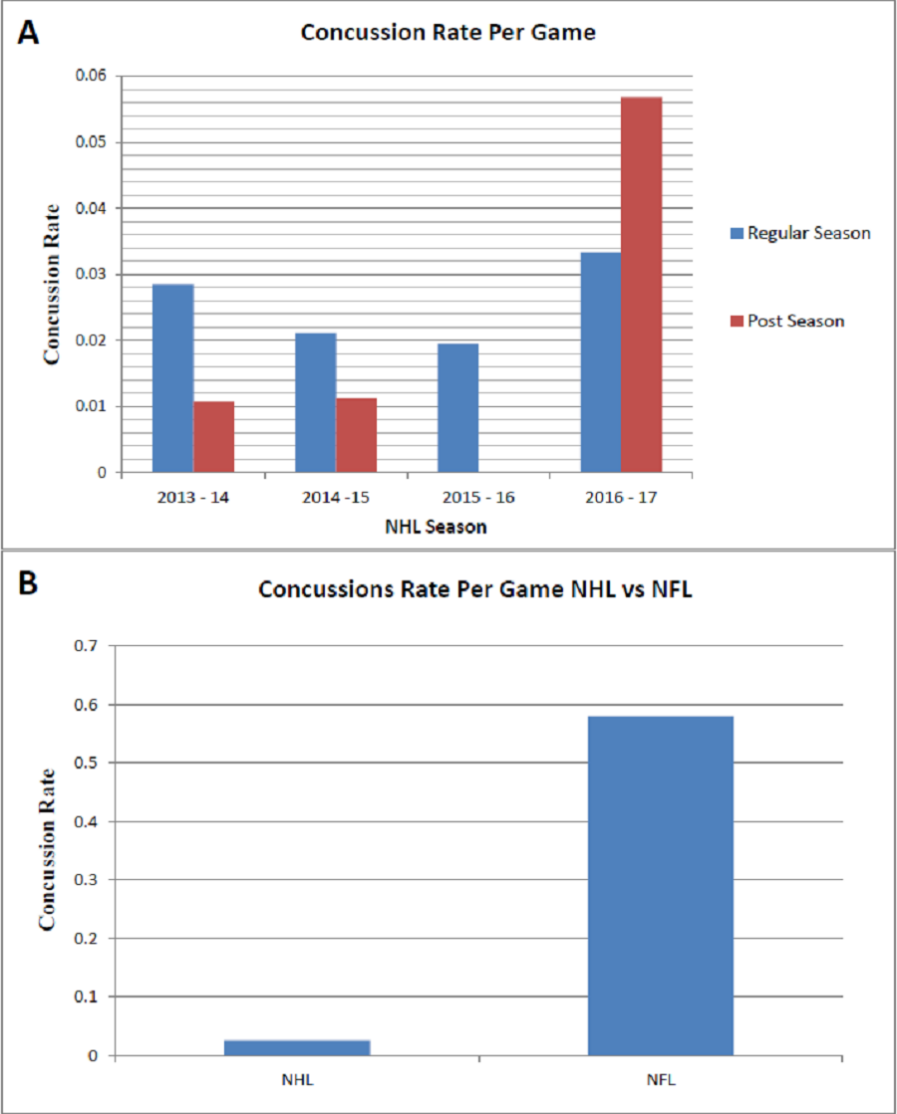
Concussion Rate Per Game Analysis (A) Concussion rate per game analyzed in both the regular season and post season for the 2013-2017 NHL seasons. No concussions were documented in the 2015-2016 post-season utilizing the FOX Sports injury tracker. (B) Concussion rate per game comparison between the National Hockey League and the National Football League. Result was ascertained utilizing the 82 game regular season plus playoffs for the NHL compared to the 16 game NFL regular seasons. NFL concussion rate was determined by our research group in a publication currently in review.

Concussion rate per game was tabulated for each individual regular and postseason game between the 2013-2017 NHL seasons (Figure 2A). Of note was the absence of any reported concussions during the 2015-2016 postseason. We also compared the concussion rate per game from our NHL dataset to a comparable study conducted by our research group which evaluated concussion rate in the National Football League (Figure 2B). The results of this analysis demonstrated a significantly reduced concussion rate per game in the NHL compared to the NFL. This result should be taken with a grain of salt due to the underreporting of concussions in our data set. NFL concussion data was derived from PBS Frontline for the 2012-2015 NFL seasons.

**Figure 3:**
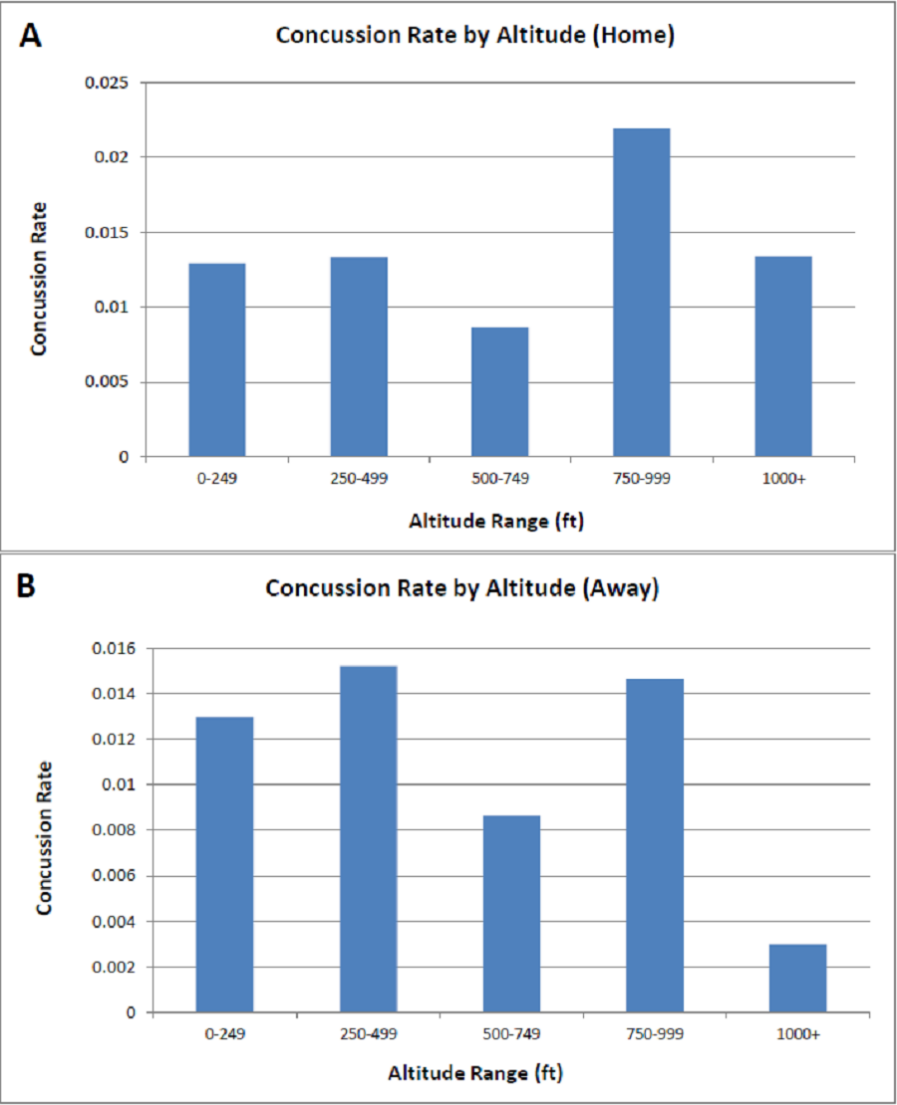
Concussion Rate by Altitude (A) Concussion rate per game tabulated by home based team and sorted by altitude ranges (B) Concussion rate per game evaluated from the away perspective and sorted by altitude ranges.

Concussion rate per game was broken down for both the home (Figure 3A) and away (Figure 3B) team altitudes into five altitude ranges to demonstrate the effect of higher altitude on concussion incidence. It should be noted that more NHL arenas are based at lower altitude compared to the total of four arenas above the 1000-foot mark.

**Figure 4:**
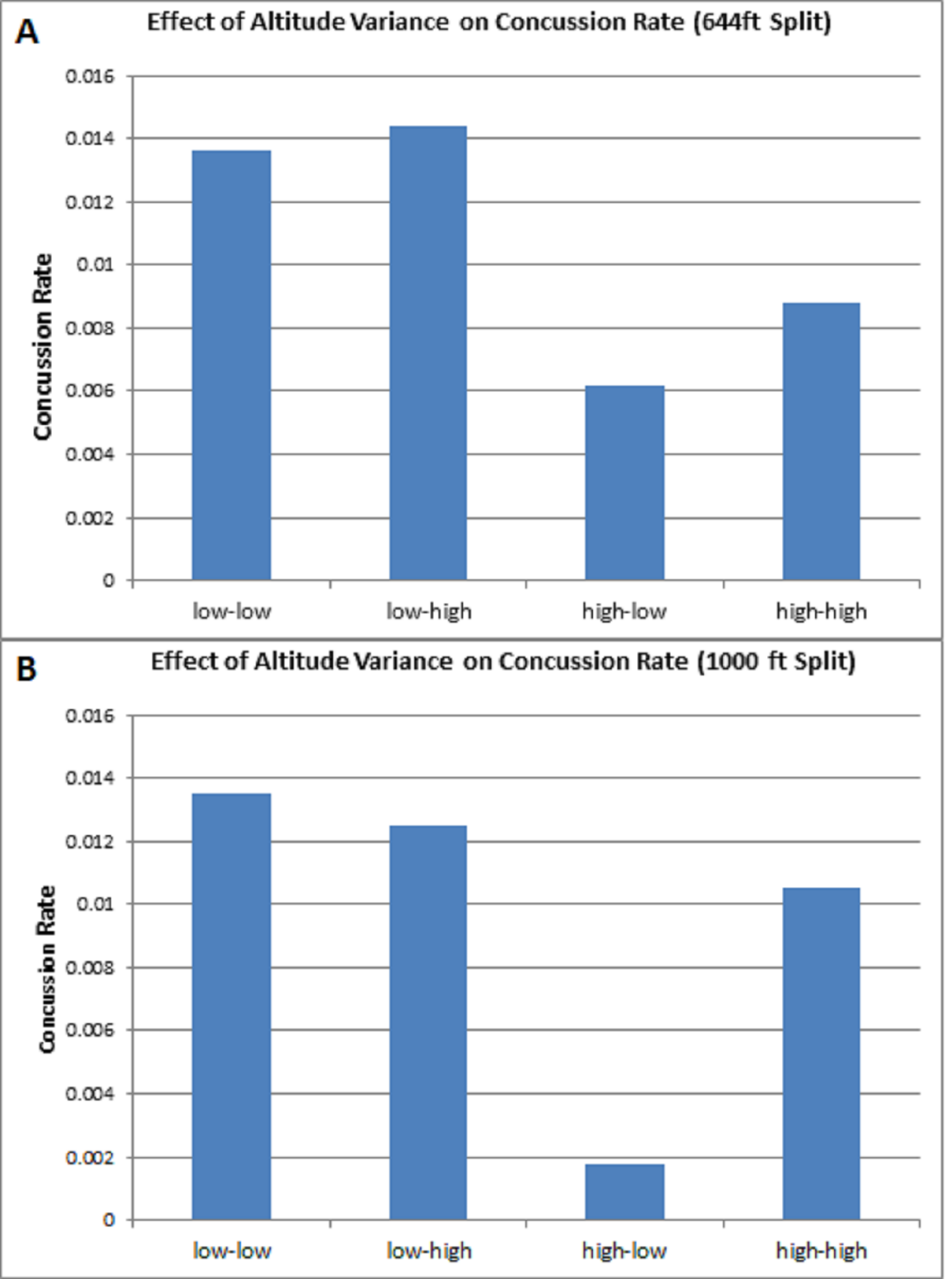
Effect of Altitude Variance on Concussion Rate (A) Game time altitude variance utilizing published low to high altitude split of 644 feet [8]. No significant difference was noted for low and high comparisons (p = 0.06288 and p=0.07186) (B) Game time altitude variance utilizing 1000 feet as the low to high altitude split. Significant difference was shown for teams based at a high altitude and traveling to a low altitude as opposed to low altitude based teams (p=0.01732 and p=0.03318). No significant difference was demonstrated for teams based and playing at high altitude (p = 0.15272).

Effect of altitude variance on concussion rate was evaluated utilizing 644 and 1000 ft as the low-high altitude split. We defined each variance by where the team is based at compared to where the game was played. This produced four distinct categories: 1) low altitude to low altitude, 2) low altitude to high altitude, 3) high altitude to low altitude, and 4) high altitude to high altitude. We noted a significant difference in concussion rate when teams based at high altitude above 1000 feet travel to play at low altitude (Figure 4B), this trend was noted in the 644 feet split although the result was non-significant (Figure 4A).

**Figure 5:**
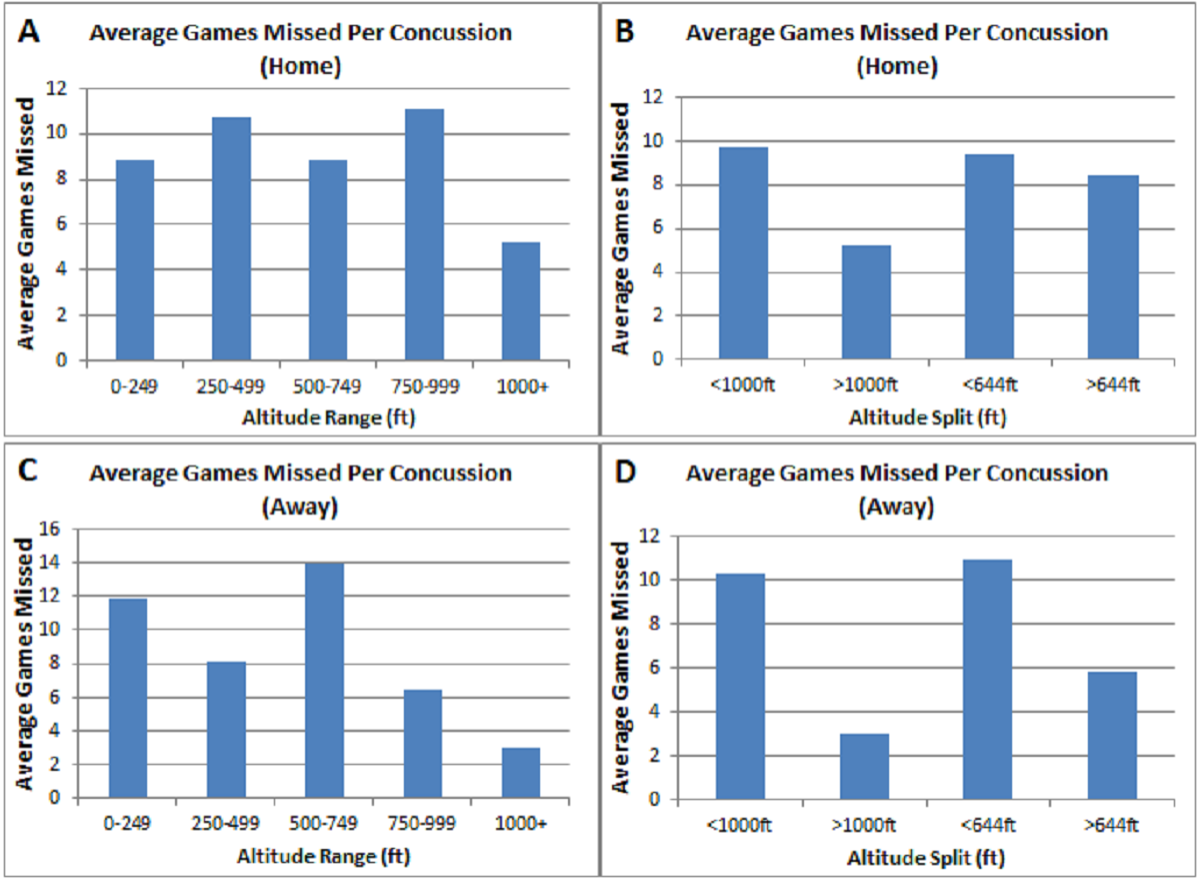
Average Games Missed Analysis (A) Average games missed per concussion from the home team perspective within altitude ranges (B) Average game missed analysis for home based teams using our identified low-high split of 1000 ft (p=0.02890) and published split of 644 ft [8] (p=0.75698) (C) Similar average games missed per concussion analysis utilizing the visiting team statistics at multiple altitude ranges. (D) Average game missed analysis for visiting teams using our identified low-high split of 1000 ft (p= 0.00087) and published split of 644 ft [8] (p=0.05944).

In order to provide a proxy for concussion severity we evaluated average games missed for both the home (Figure 5A) and away (Figure 5C) teams per concussion. This analysis was also performed utilizing the 644 and 1000 feet altitude splits. The results of this analysis demonstrated that teams that play above 1000 feet encountered fewer games missed per concussion compared to teams that are based at a low altitude for both the home and away team perspectives (Figure 5B, 5D). This significant difference was absent when evaluating average games missed using 644 ft as an altitude split (Figure 5B, 5D).

## Discussion

Greater awareness surrounding sports-related concussions has led to a rise in research pertaining to their long-term implications and potential means of mitigation. While the majority of research focuses on the mechanical effects of a concussive impact, we sought to further elucidate the role of environmental conditions, like altitude, on mean concussion rates. Our work demonstrates that teams who train and are based at a high altitude (> 1000 ft.) experience a reduction in mean concussion rates when traveling to play at a lower altitude. Interestingly, teams from low altitudes traveling and playing at high altitudes did not show the same protective benefit. Additionally, we found that the average games missed per concussion for both the home and away teams was significantly reduced for teams training at altitudes above 1000 ft. While these findings were significant, it should be noted that our data collection was extremely limited and should not be widely applied until further research is done.

The first aim of this paper was to provide more concrete data on the implications of altitude on concussions in the NHL. Current research shows conflicting data on altitude’s effect on concussion incidence. An evaluation of 6000 reported concussions in high school athletes from sea-level to roughly 7000 ft. found that increased altitude was associated with a reduced concussion rate in high school athletes [9]. Whereas a study pertaining to college football players indicated higher concussion rates at increased altitudes in conjunction with a greater proportion of athletes taking longer than six days to recover after sustaining a concussion at higher altitudes [1]. Our study suggests that there is in fact a protective advantage to longer-term training and playing at higher altitudes that helps to prevent concussions at lower altitudes. Furthermore, when concussions do occur, teams who have trained at high altitudes miss fewer subsequent games, suggesting fewer concussive symptoms and a better prognosis.

Several speculations exist to explain these findings. First, our results suggest that protective benefits against concussions are likely the result of long-term training in high altitude, rather than the direct effects of altitude itself on the brain’s recovery processes. In other words, concussion incidence might be mitigated by chronic adaptations and physiological changes of adapting to altitude long-term, rather than quick adjustments to altitudes during travel. These changes likely result from both acute increases in intracranial pressure as well as more significant long-term molecular adaptations that occur from acclimatization. These chronic adaptations seem to also lessen the symptoms when concussions do occur, as demonstrated by the fewer games missed after the incidence of an experienced concussion. In other words, our data suggests that training at high altitude both leads to a reduced incidence of concussions and a better prognosis when concussions do occur. Much of the current literature looks to explain the acute effects of altitude, but our study suggests that future attention ought to be given to the long-term effects of altitudes on the brain’s ability to cope with impact and recover.

Limitations

Our current research has several limitations. The first key limitation is the lack of reliable and publicly available data surrounding concussion incidence in the National Hockey League. Due to this drawback, our dataset should be considered as an underreported representation of the total amount of concussions spanning the 2013-2017 seasons. Additionally, there are far greater numbers of reported concussions in the NFL, which further suggests that there is probable underreporting. Thus, our data should be understood as only a sampling of the total concussions during the examined NHL seasons. A second limitation is that we did not directly take into account the schedule of each team, and thus did not compare the exact location from which each team was coming prior to a game. This limits the ability of our data to explain how sudden changes in altitude might impact concussion risk and incidence. Additionally, we did not thoroughly account for the taxing travel schedules around game days. Teams traveling between various altitudes might not have a sufficient amount of time to adequately adapt to the environmental conditions where a protective effect can be conferred. It is not uncommon for NHL teams to play back to back games in different cities, or two to three games a week with little time spent training and conditioning to each arena's altitude.

## Conclusions

Though underreported in the total number of concussions spanning the 2013-2017 NHL seasons, our data suggests that teams who train and are based at a high altitude (>1000ft) experience a reduction in mean concussion rate when traveling to play at a lower altitude and a better prognosis. In order to fully understand the impact of altitude, subsequent research should be conducted to evaluate the game-by-game travel schedules for each individual NHL team. If we are able to detail the consecutive altitudes a team plays at for multiple away games prior to returning to their home arena, it may shed further light on the acute benefits of altitude in the absence of a long-term, protective adaptation. The aim of this paper is to provide more information to professional and amateur sports organizations on the risks of concussions based on known environmental conditions. It is our goal that our findings here contribute to the larger discussion about concussion incidence and can be applied to other sports leagues and activities to mitigate their dangerous effects.
